# NFATc Acts as a Non-Canonical Phenotypic Stability Factor for a Hybrid Epithelial/Mesenchymal Phenotype

**DOI:** 10.3389/fonc.2020.553342

**Published:** 2020-09-08

**Authors:** Ayalur Raghu Subbalakshmi, Deepali Kundnani, Kuheli Biswas, Anandamohan Ghosh, Samir M. Hanash, Satyendra C. Tripathi, Mohit Kumar Jolly

**Affiliations:** ^1^Centre for BioSystems Science and Engineering, Indian Institute of Science, Bengaluru, India; ^2^Department of Clinical Cancer Prevention, UT MD Anderson Cancer Center, Houston, TX, United States; ^3^Department of Physical Sciences, Indian Institute of Science Education and Research, Kolkata, India; ^4^Department of Biochemistry, All India Institute of Medical Sciences, Nagpur, India

**Keywords:** hybrid epithelial/mesenchymal, NFATc, cancer systems biology, epithelial–mesenchymal transition, mathematical modeling, phenotypic stability factor

## Abstract

Metastasis remains the cause of over 90% of cancer-related deaths. Cells undergoing metastasis use phenotypic plasticity to adapt to their changing environmental conditions and avoid therapy and immune response. Reversible transitions between epithelial and mesenchymal phenotypes – epithelial–mesenchymal transition (EMT) and its reverse mesenchymal–epithelial transition (MET) – form a key axis of phenotypic plasticity during metastasis and therapy resistance. Recent studies have shown that the cells undergoing EMT/MET can attain one or more hybrid epithelial/mesenchymal (E/M) phenotypes, the process of which is termed as partial EMT/MET. Cells in hybrid E/M phenotype(s) can be more aggressive than those in either epithelial or mesenchymal state. Thus, it is crucial to identify the factors and regulatory networks enabling such hybrid E/M phenotypes. Here, employing an integrated computational-experimental approach, we show that the transcription factor nuclear factor of activated T-cell (NFATc) can inhibit the process of complete EMT, thus stabilizing the hybrid E/M phenotype. It increases the range of parameters enabling the existence of a hybrid E/M phenotype, thus behaving as a phenotypic stability factor (PSF). However, unlike previously identified PSFs, it does not increase the mean residence time of the cells in hybrid E/M phenotypes, as shown by stochastic simulations; rather it enables the co-existence of epithelial, mesenchymal and hybrid E/M phenotypes and transitions among them. Clinical data suggests the effect of NFATc on patient survival in a tissue-specific or context-dependent manner. Together, our results indicate that NFATc behaves as a non-canonical PSF for a hybrid E/M phenotype.

## Introduction

Metastasis remains clinically insuperable and causes over 90% of cancer related deaths (1). A hallmark of metastasizing cells is phenotypic plasticity, which empowers them to adapt to their ever-changing microenvironment, while evading therapy and immune response (2). Cells displaying phenotypic plasticity can have profound consequences: an identical genetic background can give rise to varying phenotypes under different environmental conditions, enabling non-genetic heterogeneity (3, 4), due to stochasticity in cell-fate decision making (5). A crucial axis of phenotypic plasticity during metastasis is epithelial-mesenchymal plasticity, which allows bidirectional switching of cells among an epithelial phenotype, a mesenchymal phenotype, and one or more hybrid epithelial/mesenchymal (E/M) phenotypes (6). These hybrid E/M cells can be more metastatic than cells in epithelial or mesenchymal states (7, 8) and can exhibit collective cell migration as clusters of circulating tumor cells (CTCs) (9–11) – the major drivers of metastasis (12). Thus, understanding the molecular mechanisms enabling one or more hybrid E/M phenotype(s) is key to decoding and eventually restricting metastasis.

Epithelial–mesenchymal transition (EMT) is influenced by various pathways such as transforming growth factor β (TGF-β), Wnt–β-catenin, bone morphogenetic protein (BMP), Notch, Hedgehog, and receptor tyrosine kinases (13). These EMT signals alter the levels of one or more EMT-inducing transcription factors (EMT-TFs) such as ZEB and SNAIL which can directly repress various epithelial molecules such as E-cadherin and/or induce the expression of various mesenchymal ones (6). ZEB and SNAIL form mutually inhibitory feedback loops with two microRNA families miR-200 and miR-34 where the transcription factors and the micro-RNAs mutually inhibit each other (14–17). Overexpression of ZEB promotes EMT and silences the micro-RNAs which act as a safeguard for maintaining an epithelial phenotype (14). Recent studies have indicated the involvement of phenotypic stability factors (PSF) such as GRHL2, OVOL2, NUMB, and NRF2 that can maintain the cells in a hybrid E/M phenotype(s) and prevent the cells from undergoing a complete EMT (18–23). Knockdown of these PSFs usually drove hybrid E/M cells toward a completely mesenchymal phenotype, as observed in H1975 non-small cell lung cancer (NSCLC) cells which can maintain a hybrid E/M phenotype stably over multiple passages *in vitro* (21). Higher levels of these PSFs also increased the mean residence times (MRTs) of cells in a hybrid E/M phenotype (24) and associated with poor patient survival, thus highlighting the clinical significance of hybrid E/M phenotypes (25).

Here, we investigate the role of the nuclear factor of activated T-cell (NFATc) in mediating EMT. NFATc is a family of five transcription factors (NFAT1–5), four of which (NFATc1–4) are regulated by calcium Ca^2+^ signaling (26). Initially identified as functionally important for T lymphocytes, the NFAT family regulates cell cycle progression, gene expression and apoptosis (26). Abnormalities in NFATc signaling have been reported in many carcinomas as well as lymphoma and leukemia (27). Recent evidence has suggested the interconnections of NFATc with EMT circuitry. On one hand, overexpression of NFATc increased the levels of TWIST, ZEB1, SNAI1; its downregulation decreased the levels of these EMT-TFs as well as mesenchymal markers such as N-cadherin and Vimentin (28). On the other hand, NFATc transcriptional activity was also shown to be crucial for maintaining E-cadherin levels (29), which can inhibit ZEB1 indirectly through controlling the membranous localization of β-catenin (30, 31) and restrict EMT. Moreover, NFATc can activate SOX2 (28, 32) which can upregulate levels of miR-200 (33); overexpression of miR-200 can drive mesenchymal–epithelial transition (MET) (34). These opposing interactions of NFATc with EMT circuitry lead to the question of whether NFATc promotes EMT or inhibits it. Here, we have developed a mechanism-based mathematical model that captures the interconnections between NFATc signaling and EMT circuitry. Our analysis predicts that NFATc can stabilize a hybrid E/M phenotype, facilitating cellular plasticity. Knockdown of NFATc in H1975 cells pushed the hybrid E/M cells into a mesenchymal state, validating our prediction that NFATc can function as a PSF.

## Results

### NFATc Inhibits the Progression of Complete EMT

**FIGURE 1:**
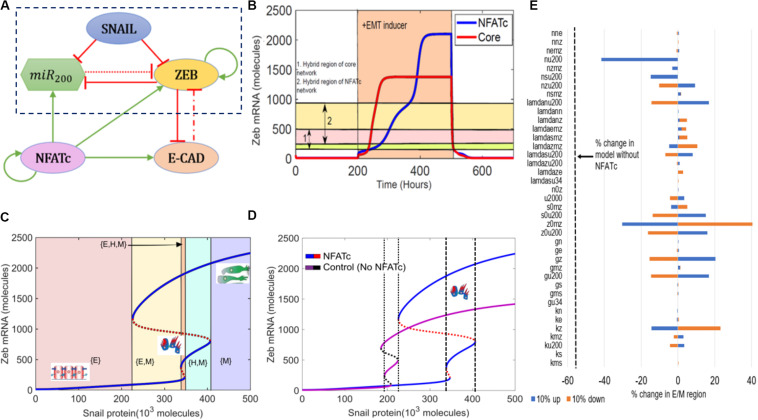
NFATc inhibits the progression of complete EMT. **(A)** Schematic representation of the EMT network coupled with NFATc. Red bars denote inhibition, green arrows indicate activation. Solid arrows represent transcriptional regulation, dash-dotted line represents indirect regulation and dotted line represent micro-RNA mediated regulation. **(B)** Temporal dynamics of ZEB mRNA levels in a cell starting in an epithelial phenotype, when exposed to a high level of *S* = 330,000 molecules (orange-shaded region) for circuit shown in **(A)** (NFATc) and that shown in the dotted rectangle in **(A)** (core). Horizontal shaded regions denote the levels of ZEB mRNA corresponding to hybrid E/M region of both NFATc (cream and pink shaded region) and the core (yellow and pink shaded region) networks. **(C)** Bifurcation diagram of ZEB mRNA levels as driven by SNAIL signal for the circuit shown in **(A)**. Curves denote the value of ZEB mRNA upon equilibration, where continuous curves stand for stable steady states and dotted lines represent unstable solutions. Different colored shaded regions show the existence/co-existence of different phenotypes; cartoons depict epithelial (E), hybrid E/M (H), and mesenchymal (M) states. **(D)** Bifurcation diagrams indicating the ZEB mRNA levels for increasing SNAIL levels for the coupled EMT-NFATc circuit (solid blue and dotted red curve) and the core EMT circuit (solid purple and dotted black curve). **(E)** Sensitivity analysis indicating percent change in the interval of SNAIL levels for stable hybrid E/M region, when corresponding parameter values are varied by ±10%. The black dotted line indicates the percent change in the stable hybrid region in the absence of NFATc (core network) when compared to the coupled network with NFATc.

To determine the role of NFATc in EMT, we first investigated the dynamics of crosstalk between NFATc and an EMT regulatory circuit (shown in the dotted rectangle) that includes miR-200, ZEB, and SNAIL (Figure 1A). This crosstalk was captured through a set of coupled ordinary differential equations (ODEs).

First, we examined the temporal dynamics of a cell in response to SNAIL levels. SNAIL represents the effect of an exogenous EMT-inducing signal such as TGF-β signaling. In the absence of NFATc, a cell that started in an epithelial state (high miR-200, low ZEB) first transitioned to a hybrid E/M phenotype and later to a mesenchymal state (low miR-200, high ZEB). The presence of NFATc, however, delayed this transition to a mesenchymal state (Figure 1B and Supplementary Figure S1A). Interestingly, the steady state value of ZEB mRNA levels was higher in case of NFATc-EMT coupled network as compared to the control case (circuit bounded by the dotted rectangle in Figure 1A); this difference can be ascribed to the activation of ZEB by NFATc (Figure 1B). Indeed, strengthening the activation of miR-200 by NFATc and/or weakening the activation of ZEB by NFATc (represented by dotted horizontal lines) prevented upregulation of ZEB levels and consequent attainment of the mesenchymal phenotype (Supplementary Figures S1B,C).

Next, we calculated a bifurcation diagram of cellular EMT phenotypes in response to increasing levels of SNAIL, an external EMT-inducing signal (Figure 1B). We observe that the system switches from epithelial to hybrid E/M and then to mesenchymal state with increasing SNAIL signal, as indicated by increasing values of ZEB mRNA and decreasing values of miR-200 (Figure 1C and Supplementary Figure S1D). Next, we compared the bifurcation diagram of the NFATc coupled network with that of the control case (i.e., without NFATc –the circuit bounded by the dotted rectangles in Figure 1A) to determine the changes in the system behavior conferred by NFATc (Figure 1D). In the presence of NFATc, a higher value of SNAIL, i.e., a stronger external signal, was required for the cells to exit the epithelial phenotype. Moreover, in the presence of NFATc, the cell maintained a hybrid E/M phenotype over a broader range of SNAIL levels (compare the range of SNAIL levels bounded by dotted lines vs. dashed lines in Figure 1D), thus requiring a much stronger stimulus to undergo a complete EMT. Stochastic simulations revealed possible cellular transitions among different phenotypes, depending on the level of SNAIL. At lower SNAIL levels, cells could possibly directly transition to a mesenchymal state from the epithelial state (Supplementary Figure S2A); however, at intermediate levels, we saw the emergence of the hybrid E/M state (Supplementary Figure S2B). At higher levels of SNAIL, the epithelial state disappears and cells can transition between the hybrid E/M and mesenchymal states (Supplementary Figure S2C).

Finally, to ascertain the robustness of the effect of NFATc in associating a larger range of SNAIL values for the existence of hybrid E/M phenotype, a sensitivity analysis was performed where each parameter of the model was varied – one at a time – by ±10%, and the corresponding change in the range of values of SNAIL enabling a hybrid E/M phenotype (i.e., the interval of *x*-axis between dashed lines) was measured. For most of the model parameters, the relative change in this range of values was quite small, suggesting the robustness of the model predictions (Figure 1E). A change in few selected parameters such as the interaction between NFATc and miR200, and self-activation of ZEB exhibited stronger sensitivity; nonetheless, even in these few cases, the decrease in range of SNAIL levels enabling a hybrid E/M phenotype is smaller when compared to the case in absence of NFATc (dotted line in Figure 1E). Put together, these observations suggest that NFATc may inhibit the progression to a complete EMT and can behave as a “phenotypic stability factor” for hybrid E/M phenotype.

### Knockdown of NFATc in H1975 Cells Promotes Complete EMT

To validate our model prediction that NFATc functions as a PSF for the hybrid E/M phenotype, we knocked down NFATc1 using siRNAs in NSCLC H1975 cells with a stable hybrid E/M phenotype.

**FIGURE 2:**
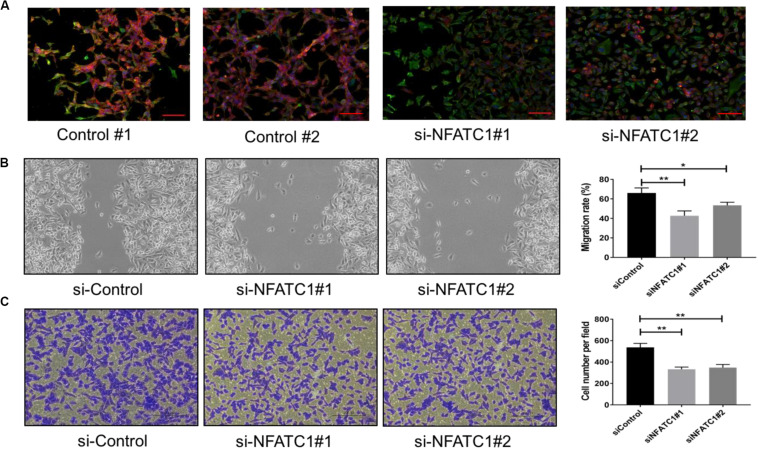
NFATc knockdown in H1975 cells promotes progression toward complete EMT. **(A)** Expression of CDH1 (E-cadherin, Red) and VIM (Vimentin, Green) examined by immuno-fluorescence staining in H1975 cells for control and NFATc1 knockdown case. Scale bar 100 μm. **(B)** Scratch assay for control H1975 cells and those treated with siRNAs against NFATc. Magnification: 100× (quantification in last column). **(C)** Same as panel **(B)** but for trans-well migration assay. *p < 0.05, **p < 0.005 using Student’s *t*-test; *n* = 3.

Individual H1975 cells can co-express E-cadherin and Vimentin (21). We observed that cells treated with NFATc1 siRNAs mostly lost E-cadherin staining (Figure 2A). NFATc knockdown decreased the E-cadherin levels and increased the levels of ZEB, SNAIL, and Vimentin, both at protein and mRNA levels (Supplementary Figures S3A–C). Thus, NFATc1 knockdown can push stable hybrid E/M cells into a mesenchymal state. Additionally, NFATc1 knockdown reduced the migration potential of H1975 cells as observed in scratch and trans-well migration assays. These findings indicate that the hybrid E/M cells may exhibit greater migratory and invasive potential when compared to mesenchymal cells (Figures 2B,C), reminiscent of our previous observations comparing hybrid E/M cells with mesenchymal ones (18). Overall, these experimental results provide a proof-of-principle validation of our model predictions that NFATc can stabilize a hybrid E/M phenotype.

### NFATc Does Not Increase the Mean Residence Time of the Hybrid E/M Phenotype

In addition to extending the range of SNAIL levels enabling a hybrid E/M phenotype, the previously identified PSFs – GRHL2, OVOL1/2, and ΔNP63α – had another trait: their presence increased the mean residence time (MRT) of cells in hybrid E/M phenotype. MRT is the average time spent by the cells in a particular phenotype (basin of attraction) – E, M, and hybrid E/M – calculated via stochastic simulations (24). Thus, the phenotype with a larger MRT implies a relatively higher stability of the same, as compared to other co-existing phenotypes/states. Hence, beyond enabling a larger range of values of SNAIL (or any other EMT inducing signal) for the existence of a hybrid E/M phenotype (as shown via bifurcation diagrams), increased MRT can be considered as another hallmark trait of a PSF. We next investigated whether NFATc increased the MRT of cells in a hybrid E/M phenotype.

**FIGURE 3:**
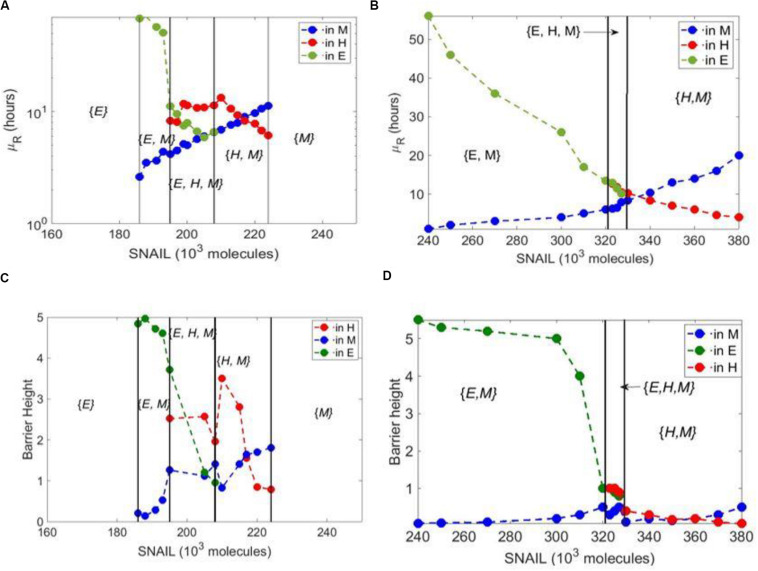
Mean residence time analysis for core EMT circuit vs. coupled EMT-NFATc circuit. **(A)** Variation of mean residence time (μ_*R*_) with varying SNAIL levels for the core circuit. **(B)** Same as panel **(A)** but for coupled EMT-NFATc circuit. **(C)** Barrier height for core EMT circuit at variable SNAIL levels. **(D)** Same as panel **(C)** but for coupled EMT-NFATc circuit.

Even though NFATc extended the range of SNAIL values enabling a hybrid E/M state, the hybrid E/M state always co-existed with epithelial and/or mesenchymal states ({E, H, M} and {H, M} phases in Figure 1B); no monostable regime ({H}) for a hybrid E/M state was seen in the case of NFATc, as observed with GRHL2, OVOL1/2, ΔNP63α, NUMB, and NRF2 (18–22, 35). Similarly, compared to the other PSFs, the presence of NFATc does not increase the absolute value of MRT for a hybrid E/M phenotype as compared to the case without NFATc. In the case of control circuit, the MRT of the epithelial state is higher than that of the mesenchymal state in the {E, M} bi-stable phase. In the {H, M} phase, the MRT of the mesenchymal state dominates that of the hybrid E/M state as SNAIL values are increased (Figure 3A). Similar trends are seen in the case of NFATc-EMT coupled network; however, in the case of {E, H, M} phase in presence of NFATc, the MRT of hybrid E/M state is not higher as compared to that of epithelial or mesenchymal states (compare the red curve in Figure 3B vs. that in Figure 3A). This trend is also seen in the barrier height calculated from the potential difference between the local minimum and saddle points corresponding to these states (Figures 3C,D).

We also plotted the potential landscapes for the NFATc-EMT coupled network at varying SNAIL levels (Supplementary Figure S4), which were consistent with the trend of barrier heights seen; for instance, at *S* = 323 × 10^3^ or *S* = 330 × 10^3^ molecules, the barrier height of hybrid E/M state was more than that of mesenchymal state, but at *S* = 380 × 10^3^, that of mesenchymal state was higher. Put together, these results suggest NFATc does not increase the MRT of hybrid E/M state.

### RACIPE Analysis of NFATc Network Reveals Its Non-canonical Behavior as a PSF

To analyze the underlying design principles of the NFATc-EMT coupled network, we employed a recently developed computational method – random circuit perturbation (RACIPE) (36). RACIPE takes as input the topology of a regulatory network and generates an ensemble of mathematical models corresponding to the network topology, each with a randomly chosen set of kinetic parameters. Then, for each mathematical model, various possible steady states (phenotypes) are identified. Finally, statistical tools are used to identify the robust dynamical properties emerging from the network topology. Here, each mathematical model is a set of five coupled ODEs, where each ODE tracks the temporal dynamics of the five species constituting the regulatory network (SNAIL, ZEB, miR200, E-CAD, and NFATc).

**FIGURE 4:**
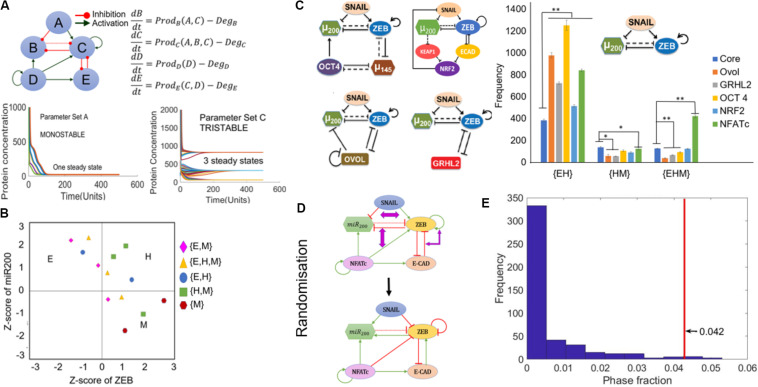
RACIPE analysis of NFATc network. **(A)** Schematic of coupled EMT-NFATc network simulated for various parametric combinations through RACIPE. For different parameters, dynamics of this regulatory network, as modeled by a set of ordinary differential equations representing the interactions in the network, can end up in a mono- or a multi-stable regime. **(B)** Phases are calculated after *z*-scores of ZEB and miR200 levels calculated from RACIPE are used to classify steady states obtained from each parameter set into Epithelial (E) – (high miR-200, low ZEB)/Hybrid (H) – (high miR200, high ZEB)/Mesenchymal (M) – (low miR-200, high ZEB) phenotypes. Different symbols denote different phases. **(C)** Frequencies of different multi-stable phases for core EMT network (SNAIL/miR-200/ZEB), its coupling to other PSFs (shown to the left of the graph) and coupled EMT-NFATc coupled network. Error bars denote standard deviation; *n* = 3. **(D)** Depiction of network randomization methodology. For every node, the degree of incoming and outgoing edges are conserved but not necessarily the number of inhibitory or excitatory edges arriving at or emanating from a node. **(E)** Frequency distribution of the {E, H, M} phase fraction for randomized networks. The red line denotes the phase fraction of {E, H, M} phase in the wild-type (WT), i.e., NFATc network. *p < 0.05, **p < 0.005 using two tailed Student’s *t*-test.

Among the 10,000 parameter sets generated via RACIPE, we found cases where the network topology can give rise to the existence of phases with one steady state (mono-stable) or more – two (bi-stable), and three (tri-stable) steady states (Figure 4A). We performed RACIPE on the core EMT network (miR-200/ZEB/SNAIL), its coupling to other PSFs (OVOL, GRHL2, OCT4, NRF2), and the coupled EMT-NFATc network. For a given parameter set, one or more steady states were obtained depending on the initial conditions chosen; each steady state solution was binned as epithelial, hybrid E/M or mesenchymal, based on the *z*-scores of miR-200 and ZEB for that case. Thus, each parameter was categorized into a given monostable or multi-stable phase; for instance, a parameter set that enabled both epithelial and hybrid E/M phenotypes for different initial conditions was classified as {E, H} (Figure 4B). Compared to the core network, each of these networks enabled a higher number of parameter sets enabling the co-existence of E and hybrid E/M states ({E, H}) (Figure 4C). Conversely, in most cases, the frequency of {H, M} (co-existence of M and hybrid E/M states) and {E, H, M} (co-existence of E, hybrid E/M, and M states) was decreased. However, in the case of NFATc, there was a significant increase in the frequency of {E, H, M} phase (Figure 4C) unlike other PSFs, suggesting that the presence of NFATc may enhance cellular plasticity among epithelial, hybrid E/M and mesenchymal states.

To test whether these results for NFATc are specific to the network topology of coupled NFATc-EMT network, we generated many randomized networks by swapping the edges between nodes in the network, such that the number of incoming and outgoing edges for every node was maintained the same (an example shown in Figure 4D). RACIPE analysis was performed on each of these randomized networks (*n* = 461; see section “Materials and Methods” for details), and the output obtained was separated into different phases i.e., {E}, {H}, {M}, {E, M}, {E, H}, {H, M}, and {E, H, M} as mentioned above. We quantified the frequency for multi-stable phases containing the hybrid state i.e., {E, H}, {H, M}, and {E, H, M} for all randomized networks, and calculated the frequencies of these phases, i.e., number of parameter sets out of 10,000 that enabled a given phase. The frequency distribution revealed that most of the randomized circuits gave rise to a lower fraction of {E, H, M} phase as compared to that for the wild-type NFATc-EMT coupled circuit (the value denoted by the red vertical line) (Figure 4E). However, such stark differences were not observed for the {E, H} (Supplementary Figure S5A) and {H, M} phases (Supplementary Figure S5B), suggesting that the NFATc-EMT coupled circuit topology is enriched for enabling co-existence and consequent possible switching among the epithelial, mesenchymal and hybrid E/M phenotypes.

### NFATc Confers Stability to the Hybrid E/M Phenotype in a Multi-Stable Phase

**FIGURE 5:**
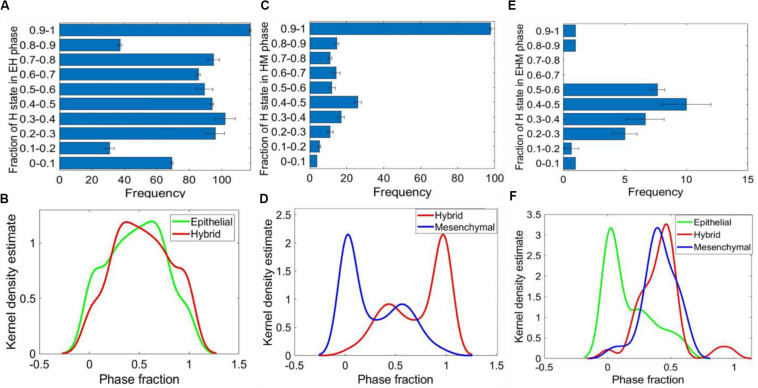
Relative stability analysis. **(A)** Frequency distribution of H state in {E, H} phase. **(B)** Kernel density plot showing the frequency distribution of E and H states in the {E, H} phase. **(C)** Frequency distribution of H state in the {H, M} phase. **(D)** Kernel density plot showing the distribution of M and H states in the {H, M} phase. **(E)** Frequency distribution of H state in the {E, H, M} phase. **(F)** Kernel density plot showing the distribution of E, H, and M states in the {E, H, M} phase. For panels **(A,C,E)** the error bars represent the mean ± standard deviation for three sets of independently chosen initial conditions for a given parameter set obtained from one RACIPE run.

We observed that the presence of NFATc in the network increased the frequency of multi-stable phases containing the hybrid state, particularly the {E, H, M} phase. This led us to investigate the relative stability of the different states in a given multi-stable phase. To quantify relative stability, every parameter set giving rise to either {E, H}, {H, M}, or {E, H, M} phases was simulated using 1000 random initial conditions and each time we tabulated how many initial conditions led to which state – E, H, or M. For individual parameter sets, we observed heterogeneity in terms of relative stability of H states in the {E, H} phase, i.e., some parameter sets seemed to have a deeper “basin of attraction” for the epithelial attractor as compared to the hybrid E/M one and *vice versa* (Figure 5A and Supplementary Figure S6A). Nonetheless, there were similarities in the frequencies of E and the H state obtained across parameter sets obtained from independent RACIPE replicates, as represented by their similar and overlapping kernel density estimates (Figure 5B and Supplementary Figures S6C,D).

This analysis for the {H, M} phase revealed that the H state was more stable; i.e., the number of parameter cases for which the relative stability of H state was higher as compared to M state was more than the number of cases when the M state was relatively more stable (Figure 5C and Supplementary Figure S5B). This trend was maintained for parameter sets obtained across three RACIPE replicates (Figure 5D and Supplementary Figures S5E,F). Similar analysis on the {E, H, M} phase suggested that the E state was relatively less stable than the H and M states (Figures 5E,F and Supplementary Figures S7A–D). Together, these results indicate that the presence of NFATc can confer high stability to the hybrid E/M state for parametric combinations enabling the co-existence of multiple phenotypes.

### NFATc Affects Clinical Outcome in a Tissue Specific Manner

**FIGURE 6:**
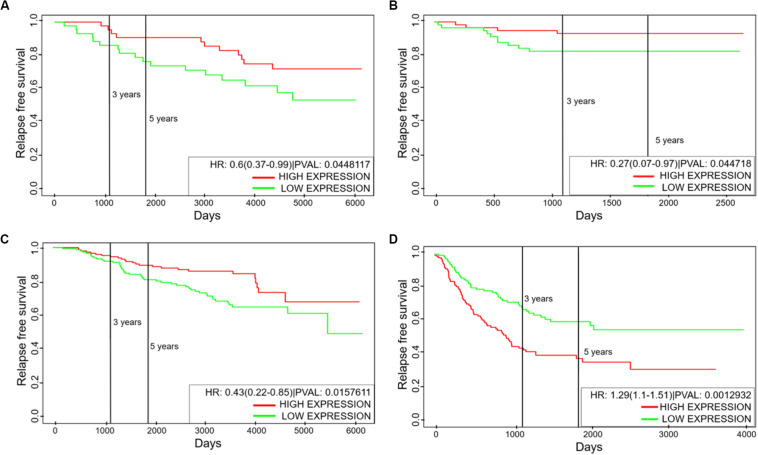
Kaplan–Meier analysis for NFATc1 levels. **(A–C)** Relapse free survival for GSE6532, GSE19615, and GSE17705 (breast cancer tissue samples). **(D)** Relapse free survival for GSE41271 (non-small cell lung cancer tissue samples). Green curve shows the group of patients with low NFATc1; red curve shows the group with high NFATc1 levels. All cohorts are divided at median values of NFATc1; HR denotes hazard ratio; PVAL denotes *p*-value.

The hybrid E/M phenotype is often attributed to drive tumor aggressiveness (7, 8). This trend is further supported by clinical data where PSFs such as GRHL2 and NRF2 correlate with poor patient survival (18, 21). We investigated whether the levels of NFATc can correlate with clinical response, and observed that the association of NFATc with clinical outcomes is context dependent. Higher levels of NFATc correlated with better relapse free survival among breast cancer patients (Figures 6A–C) but with poor relapse free survival among lung cancer patients (Figure 6D). We observed similar context-dependent behavior of NFATc in terms of overall survival, even within the same tissue (Supplementary Figures S8A–C). Also, NFATc was positively correlated with better metastasis free survival among breast cancer patients (Supplementary Figure S8D). Thus, the correlation of NFATc with patient survival is highly likely to be context dependent.

## Discussion

Recent *in vitro*, *in vivo*, and *in silico* investigations have emphasized the existence and significance of hybrid E/M phenotype(s) in various cancer types (37). These hybrid E/M phenotypes can exhibit maximum plasticity (38), possess traits of cancer stem cell-like traits, evade drug resistance, and thus be the “fittest” for metastasis (8). These preclinical experimental observations are supported by clinical analysis of carcinoma samples suggesting that the presence of hybrid E/M cells in a patient at the time of diagnosis associates with poor patient outcomes. Interestingly, even a very small percentage of hybrid E/M cells (score >2%) was found to be sufficient to confer poor prognosis (39). Thus, identifying mechanisms that can maintain cells in hybrid E/M phenotypes is of crucial importance in our efforts to curb metastatic load.

Here, we developed a computational modeling framework to identify the transcription factor NFATc as a potential PSF for hybrid E/M phenotypes. In presence of NFATc, cells undergo a delayed or stalled EMT; thus maintaining cells in hybrid E/M phenotypes; knockdown of NFATc in H1975 NSCLC cells drove the progression toward a complete EMT phenotype, reminiscent of observations made for other PSFs – GRHL2, OVOL2, NUMB, and NRF2 (18–23). Similar effects of NFATc knockdown were also seen in MCF10A and DLD1 cells where treatment with VIVIT, a soluble inhibitor of NFATc transcriptional activity, significantly reduced E-cadherin expression and protein level, and increased Slug and Vimentin levels (29), thus driving EMT. NFATc transcriptional activity was shown to be capable of maintaining E-cadherin levels even in the presence of TGFβ induced EMT (29), suggesting that NFATc acted as a “molecular brake” or “guardian” of epithelial traits, preventing a complete EMT (40). Consistently, NFATc1 was identified to be a master regulator of chromatin remodeling to regulate hybrid E/M phenotypes in skin cancer *in vivo*; the proportion of hybrid E/M phenotypes was also shown to be increased by GRHL2, OVOL1/2, and ΔNP63α at the expense of complete EMT cells, thus lending further credence to our results indicating a functional equivalence between NFATc1 and previously identified PSFs such as GRHL2, OVOL1/2, ΔNP63α, and NRF2 (7). In developmental EMT scenario, NFATc1 is implicated in a key role during heart valve development; NFATc1-null embryos exhibit excessive EMT and impaired valve formation. Transcriptional repression of Snail1 and Snail 2 by NFATc1 can inhibit EMT and help maintain vascular E-cadherin levels required for cellular adhesiveness (41, 42). These observations across multiple contexts highlight that NFATc may maintain cell-cell contacts in a hybrid E/M phenotype.

Hybrid E/M phenotype(s) are also often associated with higher stem-like behavior and enhanced metastasis across cancer types *in vitro* and *in vivo* (25). Consistently, NFAT transcriptional activity contributes to metastasis in colon cancer; inhibition of NFATc1 reduced metastatic growth in an immunocompetent mouse model. Further, genes upregulated by NFATc1 significantly correlated with worse clinical outcomes for Stage II and III colorectal cancer patients (43). Similarly, NFATc2 was overexpressed in lung adenocarcinoma tumor-initiating cells; it supported tumorigenesis *in vivo* and its knockdown *in vitro* reduced 60–70% tumor-spheres and restricted the renewability of tumor-spheres (32). NFAT/calcineurin signaling pathway is also activated in breast cancer and aggravates tumorigenic and metastatic potential of mammary tumor cells *in vitro* and *in vivo* (44, 45). Furthermore, NFATc1 levels were found to be significantly upregulated in spheroid-forming cells in pancreatic cancer, where NFATc1 promotes SOX2 transcriptionally (28). One of the targets of NFAT/SOX2 signaling pathway is ALDH1A1 (32) – a *bona fide* marker for hybrid E/M cells behaving as cancer stem cells in breast cancer (46). Therefore, these observations underscore the connection between NFAT signaling, stemness, and metastatic aggressiveness.

Our results show that while NFATc increased the parametric range of SNAIL levels enabling a hybrid E/M phenotype, it did not increase the MRT of hybrid E/M cells, suggesting that the role of NFATc may be non-canonical in terms of behaving as a PSF. This non-canonical behavior is further elucidated by RACIPE analysis, where, unlike other PSFs such as GRHL2, NFATc increased the frequency of parametric combinations containing co-existing epithelial, hybrid E/M and M phenotypes, and possible interconversions among them. Thus, NFATc may be thought of as a driver of phenotypic plasticity, and targeting NFAT signaling may curb cancer cell adaptation (47) – a distinctive property of metastasis-initiating cells (48). Given that at least a full-blown EMT by itself need not be necessary for metastasis, the emergent dynamics of metastatic networks (49, 50) can also hold clues for identifying other perturbations to curb metastatic load.

## Materials and Methods

### Mathematical Modeling

As per the schematic shown in Figure 1A, the dynamics of all five molecular species (miR-200, SNAIL, ZEB, E-cadherin, and NFATc) was described by a system of coupled ODEs. The level of a protein, mRNA or micro-RNA (X) is described via a chemical rate equation that assumes the generic form:

$$\frac{d\,X}{d\,t}=g_{X}\,H^{S}\,\left(A,A_{0},n,\lambda \right)-k_{X}\,X$$

Where the first term of the equation signifies the basal rate of production (*g*_*X*_); the terms multiplied to *g*_*X*_ represent the transcriptional/translational/post-translational regulations due to interactions among the species in the system, as defined by the Hills function [*H^S^*(*A*,*A*_0_,*n*,λ)]. The term *k*_*X*_*X* accounts for the rate of degradation of the species (X) based on first order kinetics. The complete set of equations and parameters are presented in the Supplementary Material.

### Cell Culture and siRNA Treatments

H1975 cells were cultured in RPMI 1640 medium containing 10% fatal bovine serum and 1% penicillin/streptomycin cocktail (Thermo Fisher Scientific). Cells were transfected at a final concentration of 50 nM siRNA using Lipofectamine RNAiMAX (Thermo Fisher Scientific) according to the manufacturer’s instructions using following siRNAs: siControl (Thermo Fisher Scientific), siNFATC1 #1 (Invitrogen), siNFATC1 #2 (Invitrogen). Regular mycoplasma testing was also carried out to exclude any possible cell culture contamination.

### RT-PCR

Total RNA was isolated following manufacturer’s instructions using RNAeasy kit (Qiagen). cDNA was prepared using iScript gDNA clear cDNA synthesis kit (Bio-Rad). A TaqMan PCR assay was performed with a 7500 Fast Real-Time PCR System using TaqMan PCR master mix, commercially available primers, and FAM^TM^-labeled probes for CDH1, VIM, ZEB1, NFATC1, SNAIL, and VIC^TM^-labeled probes for 18 S, as per manufacturer’s instructions (Life Technologies). Each sample was run in biological and technical triplicates. Ct values for each gene was calculated and normalized to Ct values for 18 S (ΔCt). The ΔΔCt values were then calculated by normalization to the ΔCt value for control.

### Western Blotting Analysis

H1975 cells were lysed in RIPA lysis assay buffer (Pierce) supplemented with enzyme inhibitor cocktail (Roche). The samples were separated on a 4–20% SDS-polyacrylamide gel (Bio-Rad). After transfer to PVDF membrane, incubation was carried out with primary antibodies anti-CDH1 (1:1000; Cell Signaling Technology), anti-vimentin (1:1000; Cell Signaling Technology), anti-Zeb1 (1:1000; Cell Signaling Technology), anti-SNAIL (1:1000; Cell Signaling Technology), and anti-beta actin (1:10 000; Abcam) and subsequent secondary antibodies. Membranes were exposed using the ECL method (GE Healthcare) as per manufacturer’s instructions.

### Immunofluorescence

H1975 Cells were fixed in 3.4% paraformaldehyde, permeabilized with 0.2% Triton X-100, and then stained with primary antibodies against CDH1 (1:100; Abcam) and vimentin (1:100; Cell Signaling Technology). Alexa conjugated secondary antibodies (Life Technologies) were used to detect the expression of respective proteins. DAPI was used to counterstain the nuclei.

### Wound-Healing Assay

Scratch wound-healing assay was performed to determine cell migration using confluent cultures (80-90% confluence). Briefly, H1975 cells (1 × 105 cells/ml) were seeded in 6-well tissue culture plate. After cells attain expected confluency, they were starved for 24 h using 0.2% serum in growth media. Next day, a sterile p200 pipet tip was used to create a wound on the confluent monolayer and media was replenished. Images were acquired at 0 and 16 h; the experiments were repeated three times. Images of the scratch wounds were taken and measured by ImageJ software to calculate the mean and standard deviation. Each group was compared with the control group. Cell migration was expressed as the migration rate: (original scratch width – new scratch width)/original scratch width × 100%.

### Trans-Well Migration Assay

H1975 cells were grown in 6-well plates and treated with siNFATC1 for 24 h. After 48 h of NFATC1 knockdown, cell monolayers were harvested, and 2 × 104 viable cells/200 μl cell concentration was prepared in serum free medium. The cell suspension was transferred on top of a 0.8 μm pore diameter Transwell insert (Millipore) and placed on a 24 well cell culture plate. A 10% fetal bovine serum solution was added as chemo-attractant at the bottom of the insert and plate was incubated at 37°C for 18 h. Non-migrated cells were removed by gently swabbing inside each insert. Cells were fixed and stained with a 0.5% crystal violet solution for 10 min. The inserts were thoroughly washed with and air dried completely before visualizing under a microscope. Cell numbers were counted at ×200 magnification. The experiment was repeated three times and statistically analyzed with five fields of view, and the mean values were taken as the migratory cell number.

### Mean Residence Time Analysis

The MRT was calculated as follows: As the degradation rate of ZEB mRNA is much greater than that of ZEB protein and miR-200 and also the production rate of E-cad and NFATc is much more larger than that of ZEB protein and miR-200, we assumed that ZEB mRNA, E-cad, and NFATc reach to the equilibrium much faster relatively, that is, $\frac{d\,m_{Z}}{d\,t}=0$, $\frac{d\,E}{d\,t}=0$, and $\frac{d\,N}{d\,t}=0$. This assumption reduces the equations given in Supplementary Material: to two coupled ODEs of ZEB and miR-200. Then we simulated the dynamical system in presence of external noise and obtained the time evolution of ZEB protein and miR-200 using Euler–Maruyama simulation. From the time evolution of ZEB and miR-200 the dynamical states of the system were coarse grained as an itinerary of basins visited. Then the MRT was calculated by multiplying the total number of successive states with Δt. Detailed methods are outlined in the publication by Biswas et al. (24).

### RACIPE

Random circuit perturbation (36) algorithm was run on the coupled EMT-NFATc network and its randomized counterparts. Continuous steady state levels were obtained as output for the five variables, for ensembles of mathematical models; each model has a randomly chosen parameter set corresponding to intrinsic production/degradation of all species as well as those representing the regulatory links. The algorithm was used to generate 10,000 mathematical models, each with a different set of parameters. Hundred initial conditions were chosen for each model, and all steady state solutions obtained were compiled together. With this consolidated data, the *z*-scores of steady state levels of all the biomolecules in the individual networks were calculated. Based on the *z*-score of ZEB and miR-200, the phenotype for a given steady state solution is decided, i.e., if (z_*zeb*_ > 0) and (z_*mir–*200_ < 0), it is counted as mesenchymal state, (z_*zeb*_ > 0) and (z_*mir–*200_ > 0) is counted as a hybrid state, and (z_*zeb*_ < 0) and (z_*mir–*200_ > 0) is counted as an epithelial state. Similarly, states are determined for all solutions and based on the state of each steady state for a given set of parameters, the phases are determined.

### Network Randomization

The following rules were employed to generate an ensemble of randomized networks. For each node, in each instance of a randomization of the wild-type network (Figure 1A), the number of incoming and outgoing edges were kept constant. The number of activation edges and the number of inhibitory edges were also kept fixed at 6 and 5, respectively [The same number as that in the wild-type network (Figure 1A)]. Furthermore, the source node and the target node for each of the edges were kept fixed but the identity of the edge in terms of it being an activation or inhibition link was allowed to change. Hence, 461 (${}_{5}^{11}\,C-1=\frac{11!}{6!\,5!}-1=461$) such randomized networks were constructed excluding the wild-type case.

### Relative Stability Analysis

The relative stability analysis was performed in MATLAB. RACIPE generated parameter sets that give rise to multi-stable phases were first determined. These parameter sets were simulated in MATLAB using 1000 random initial conditions and the steady state each time was determined. Then, the total number of times each of the possible steady states was reached was calculated.

### Kaplan–Meier Analysis

ProgGene (51) was used for conducting Kaplan–Meier analysis for respective datasets. The number of samples in NFATc1-high vs. NFATc1-low categories are given below:

GSE6532 (breast cancer): *n* (High) = 44, *n* (Low) = 43

GSE19615 (breast cancer): *n* (High) = 58, *n* (Low) = 57

GSE17705 (lung cancer): *n* (High) = 149, *n* (Low) = 149

GSE41271 (lung cancer): *n* (High) = 138, *n* (Low) = 137

GSE30219 (lung cancer): *n* (High) = 141 *n* (Low) = 141

GSE19536 (breast cancer): *n* (High) = 56, *n* (Low) = 44

GSE14814 (lung cancer): *n* (High) = 44, *n* (Low) = 44.

## Data Availability Statement

The datasets presented in this study can be found in online repositories. The names of the repository/repositories and accession number(s) can be found in the article/Supplementary Material.

## Author Contributions

MKJ and ST designed the research. MKJ, ST, AG, and SH supervised the research. ARS, DK, and KB performed the research. All authors contributed to analyzing the data and writing and editing of the manuscript.

## Conflict of Interest

The authors declare that the research was conducted in the absence of any commercial or financial relationships that could be construed as a potential conflict of interest.
