# Supplementary material for: NFATc Acts as a Non-Canonical Phenotypic Stability Factor for a Hybrid Epithelial/Mesenchymal Phenotype
Source: Front Oncol. 2020 Sep 8;10:553342. doi: 10.3389/fonc.2020.553342 (PMC7506140; doi:10.3389/fonc.2020.553342)
Supplement: Supplementary file 1 [file Data_Sheet_1.docx]

**NFATc acts as a non-canonical phenotypic stability factor**

**for a hybrid epithelial/mesenchymal phenotype**

**Supplementary Information**

**Mathematical modelling**

The dynamics of the NFATc-EMT coupled circuit describes the dynamics of the molecular species of the EMT regulatory circuit (miR-200, Snail, Zeb), E-cadherin and NFATc as shown in Fig 1A. This set-up extends the mathematical model of EMT circuit previously developed (1). The given set of coupled ordinary differential equations (ODEs) represent the dynamics of the species of the circuit (miR-200: μ_200_, Snail: S, Zeb: Z, E-cadherin: E, NFATc: N):

$\frac{{d\mu}_{200}}{dt}= g_{\mu_{200}}H^{s}$(Z,$\lambda_{{Z,\mu}_{200}})H^{s}\left( S,\lambda_{{S,\mu}_{200}} \right)H^{s}\left( N,\lambda_{{N,\mu}_{200}} \right)- m_{Z}Y_{\mu}\left( \mu_{200} \right)- k_{\mu_{200}}\mu_{200}$

$\frac{{dm}_{Z}}{dt}= g_{m_{Z}}H^{s}$(Z,$\lambda_{{Z,m}_{Z}})H^{s}\left( S,\lambda_{{S,m}_{Z}} \right)H^{s}\left( N,\lambda_{{N,m}_{Z}} \right)H^{s}\left( E,\lambda_{{E,m}_{Z}} \right)- m_{Z}Y_{Z}\left( \mu_{200} \right)- k_{m_{Z}}m_{Z}$

$$\frac{dZ}{dt}= g_{Z}m_{Z}L(\mu_{200})- k_{Z}Z$$

$\frac{dE}{dt}= g_{E}H^{s}$(Z,$\lambda_{Z,E})H^{s}$(N,$\lambda_{N,E})- k_{E}E$

$\frac{dN}{dt}= g_{N}H^{s}$(N,$\lambda_{N,N})- k_{N}N$

where g_X_ is the corresponding innate production rate and k_X_ is the innate degradation rate.

m_z_L(μ_200_) is the net translation rate, m_z_Y_m_(μ_200_) is the total ZEB mRNA active degradation rate and m_z_Y_μ_(μ_200_) is the total miR active degradation rate. H^S^ is the shifted Hill function, defined as

H^S^(B,λ ) = H^−^(B)+λH^+^(B) ,

H^−^(B) =1/ [1+(B / B_0_)^nB^ ] ,

H^+^(B) =1−H^−^ (B),

λ is the fold change from the basal synthesis rate due to protein B. λ >1 for activators, while λ<1 for inhibitors.

**Parameter estimation**

The parameters were adopted from previously published literature for the molecular species of the EMT regulatory circuit (miR-200, Snail, Zeb), E-cadherin and NFATc interactions, as given below:

| **Parameter** | **Value** | **Reference** |
| --- | --- | --- |
| $g_{\mu_{200}}$(Molecules/Hour) | 2.1K | (1) |
| $g_{m_{Z}}$(Molecules/Hour) | 11 | (1) |
| Z^0^μ_200_ (Molecules) | 220K | (1) |
| Z^0^m_Z_ (Molecules) | 25K | (1) |
| $n_{{Z,\mu}_{200}}$ | 3 | (1) |
| $n_{{Z,m}_{Z}}$ | 2 | (1) |
| $n_{\mu_{200}}$ | 6 | (1) |
| $n_{{S,\mu}_{200}}$ | 2 | (1) |
| $n_{{S,m}_{Z}}$ | 2 | (1) |
| $\lambda_{{Z,\mu}_{200}}$ | 0.1 | (1) |
| $\lambda_{{Z,m}_{Z}}$ | 7.5 | (1) |
| $\lambda_{{S,\mu}_{200}}$ | 0.1 | (1) |
| $\lambda_{{S,m}_{Z}}$ | 10 | (1) |
| $k_{\mu_{200}}$(Hour^-1^) | 0.05 | (1) |
| $k_{m_{Z}}$(Hour^-1^) | 0.5 | (1) |
| $k_{Z}$(Hour^-1^) | 0.1 | (1) |
| $g_{Z}$(Hour^-1^) | 0.1K | (1) |
| $S_{\mu_{200}}^{0}$(Molecules) | 180K | (1) |
| $S_{m_{Z}}^{0}$(Molecules) | 180K | (1) |
| S(Molecules) | 200K | (1) |
| $\mu_{200}^{0}$(Molecules) | 10K | (1) |
| $g_{E}$(Molecules/Hour) | 5000 | (2) |
| $k_{E}$(Hour^-1^) | 0.1 | (2) |
| $\lambda_{{E,m}_{Z}}$ | 0.8 | (2) |
| $n_{{E,m}_{Z}}$ | 2 | (2) |
| $E_{m_{Z}}^{0}$(Molecules) | 80000 | (2) |
| $\lambda_{Z,E}$ | 0.1 | (2) |
| $n_{Z,E}$ | 2 | (2) |
| $Z_{E}^{0}$ | 100000 | (2) |
| $\lambda_{N,E}$ | 8 | (3) |
| $n_{N,E}$ | 5 | (4) |
| $N_{E}^{0}$ | 100000 | Estimated |
| $g_{N}$(Molecules/Hour) | 80000 | (4) |
| $k_{N}$(Hour^-1^) | 0.1 | (4) |
| $\lambda_{N,Z}$ | 2 | (4) |
| $n_{N,Z}$ | 2 | (4) |
| $N_{Z}^{0}$ | 800000 | Estimated |
| $\lambda_{{N,\mu}_{200}}$ | 4 | Estimated |
| $n_{{N,\mu}_{200}}$ | 4 | Estimated |
| N^0^_μ200_ (Molecules) | 500000 | Estimated |
| $\lambda_{N,N}$ | 7 | (5) |
| $n_{N,N}$ | 3 | (5) |
| $N_{N}^{0}$ | 100000 | Estimated |

K denotes 10^3^ molecules

**Sensitivity analysis**

The data presented in Fig 1E is presented in Table S1. For an increase or decrease by 10%, one parameter at a time, the bifurcation diagrams were plotted and numerical values of limit points were identified to quantify the degree of changes in the range of SNAIL levels for which hybrid E/M branch was present in bifurcation diagram in a mono/multi-stable phase.

**Supplementary Figures**


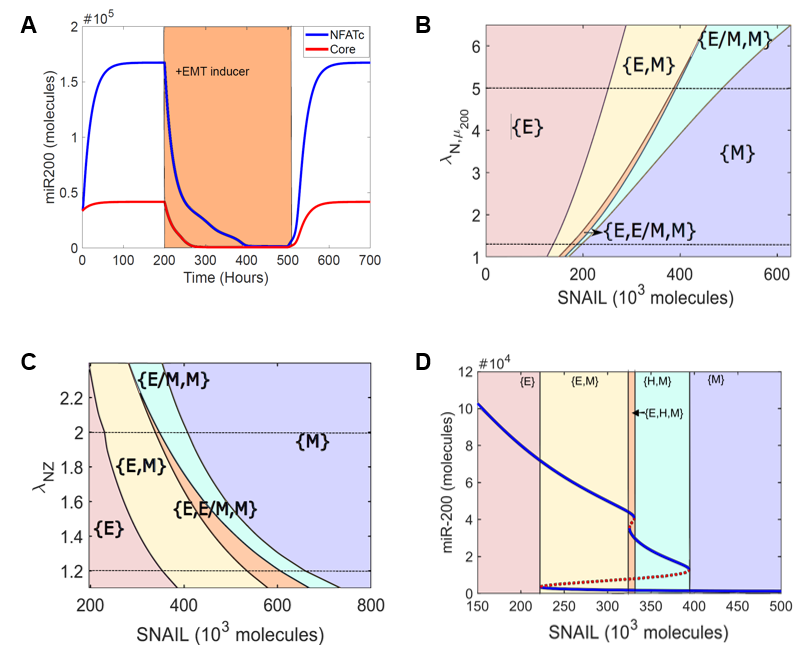


***FigS1: NFATc inhibits a complete EMT*** *A) Dynamics of miR200 levels in a cell starting in an epithelial phenotype, when exposed to a high level of S=330000 molecules (orange-shaded region). B) Phase diagram of NFATc network when driven by SNAIL and varying strength of interaction between NFATc and miR200. The dotted line at the top represents NFATc over-expression leading to stronger interaction between NFATc and miR200. The dotted line at the bottom represents NFATc knock-down resulting in a weaker interaction between NFATc and miR200. C) Phase diagram of NFATc network when driven by SNAIL and varying strength of interaction between NFATc and ZEB. The dotted line at the top represents NFATc over-expression leading to stronger interaction between NFATc and ZEB. The dotted line at the bottom represents NFATc knock-down resulting in a weaker interaction between NFATc and ZEB. D) Bifurcation diagram of miR200 levels as driven by SNAIL signal. Solid blue curves show stable states, dotted red lines show unstable states. Different coloured regions show different combinations of phases (co-existing states).*

To quantify the effect of NFATc activating ZEB and/or miR-200, we plotted phase diagrams by varying the strength of activation of ZEB1 and that of miR-200 (Fig S1B,C) by NFATc. When NFATc activates ZEB1 weakly (lower dotted line in Fig S1B), the monostable M ({M}) phase is attained at a lower value of SNAIL as compared to the case when NFATc activates ZEB1 strongly (upper dotted line in Fig S1B). Consistently, an opposite trend is seen in case of NFATc activating miR-200 (dotted lines in Fig S1C).


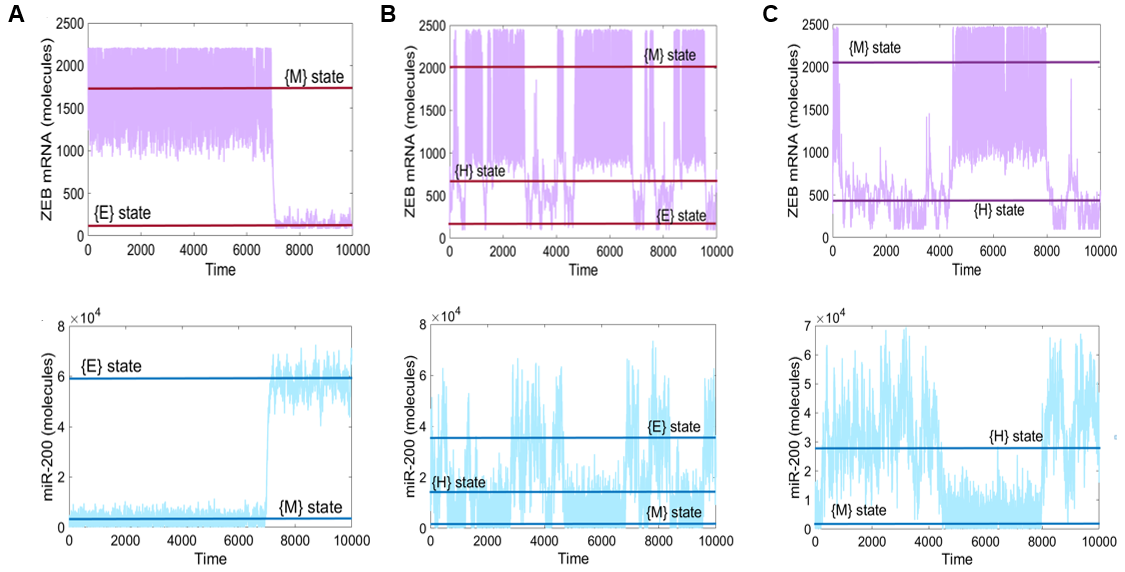


***FigS2: Stochastic simulations for NFATc network.*** *A) At S=260K, the cells switch between E and M states. The top panel in purple represents the level of ZEB mRNA molecules and the bottom panel in blue represents the levels of miR200 levels. B) Same as A) but for S=325K, where cells can switch among E, H and M states. C) Same as A) but for S=330K where cells can switch among H and M states.*


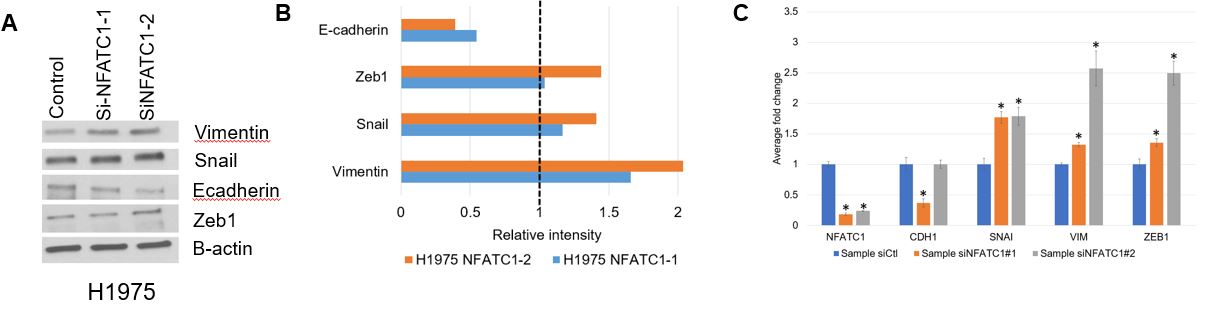


***FigS3: Knockdown of NFATc drives a complete EMT.*** *A) Quantification of western blot analysis shows decreased levels of E-CAD and increased levels of VIM, SNAIL and ZEB with knockdown of NFATc1. B) Quantitative RT-PCR for CDH1, VIM, SNAIL, ZEB1 and NFATc1before and after treatment with siRNAs against NFATc1. C) Western blot for CDH1, VIM, ZEB1, SNAIL in H1975 cells. β-actin is used as loading control. Left column represents the control siRNA, second and third columns represent two independent siRNAs against NFATc1. *, p < = 0.01 using two tailed t-test.*


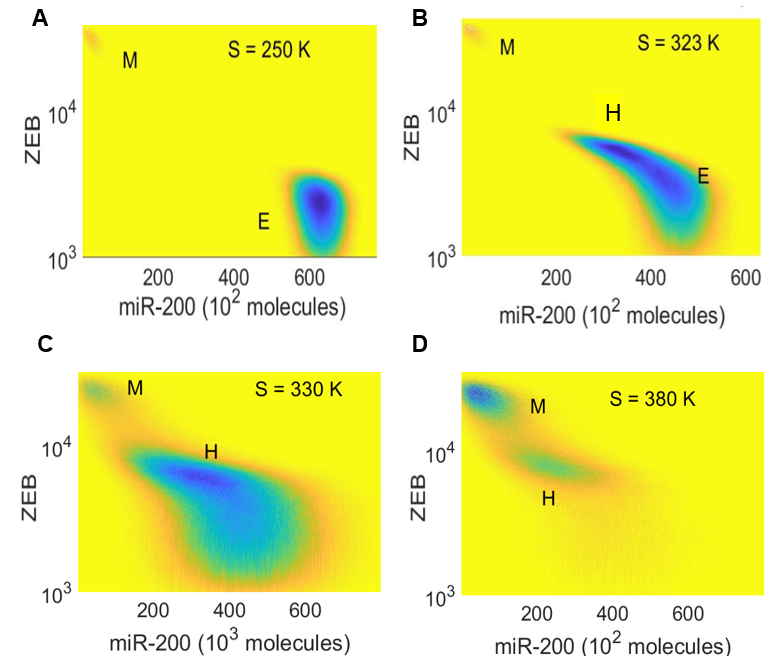


***FigS4: Potential landscape for NFATc-EMT coupled circuit at varying SNAIL levels***


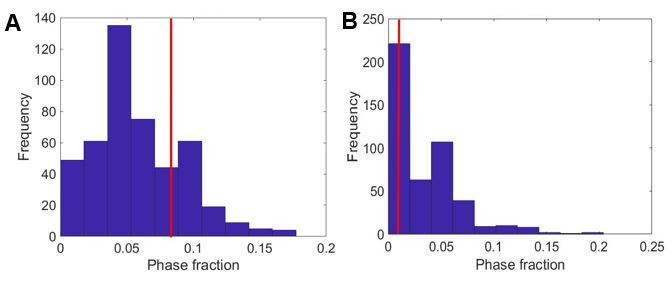


***FigS5: Phase analysis of randomized networks.*** *A) Frequency distribution of {E, H} phase fraction for 561 randomized networks. The red line denotes the phase fraction of {E, H} phase in the wild type (WT) NFATc-EMT coupled network. B) Frequency distribution of {H, M} phase fraction for 561 randomized networks. The red line denotes the phase fraction of {H, M} phase in the wild type (WT)*


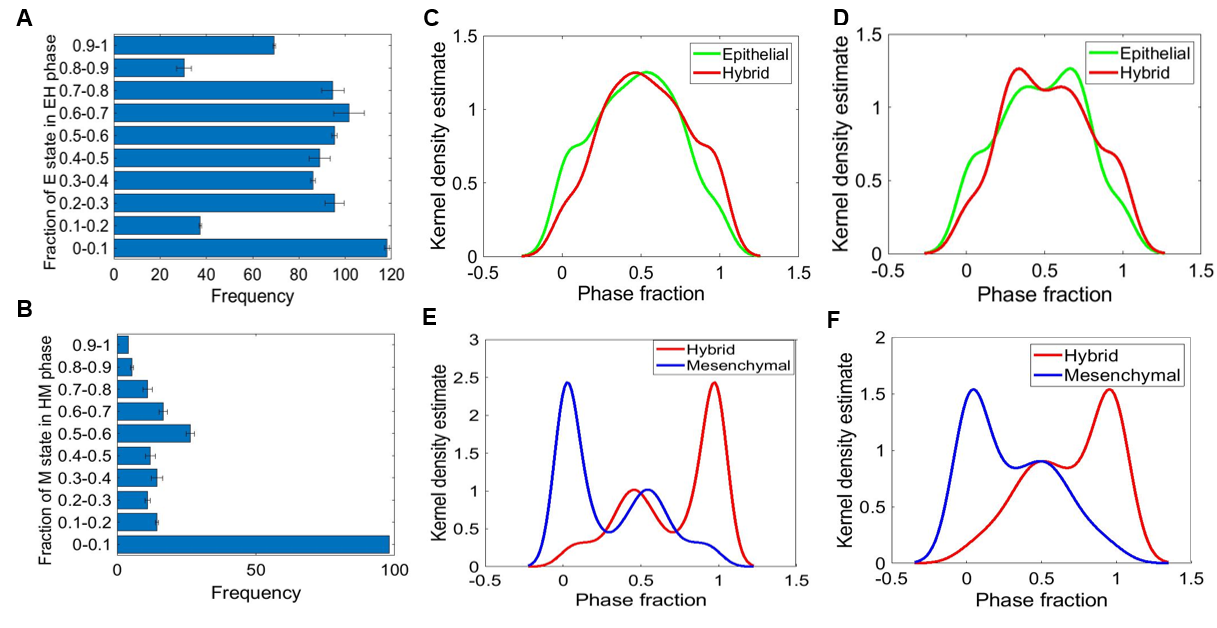


***FigS6: Relative stability analysis.*** *A) Frequency distribution of E state in {E,H} phase. B) Frequency distribution of M state in {H,M} phase. C-D) Density plots showing the distribution of E and H state in the {E,H} phase for two parameter sets obtained from independent RACIPE replicates. E-F) Same as C-D but for H and M states in the {H, M} phase.*


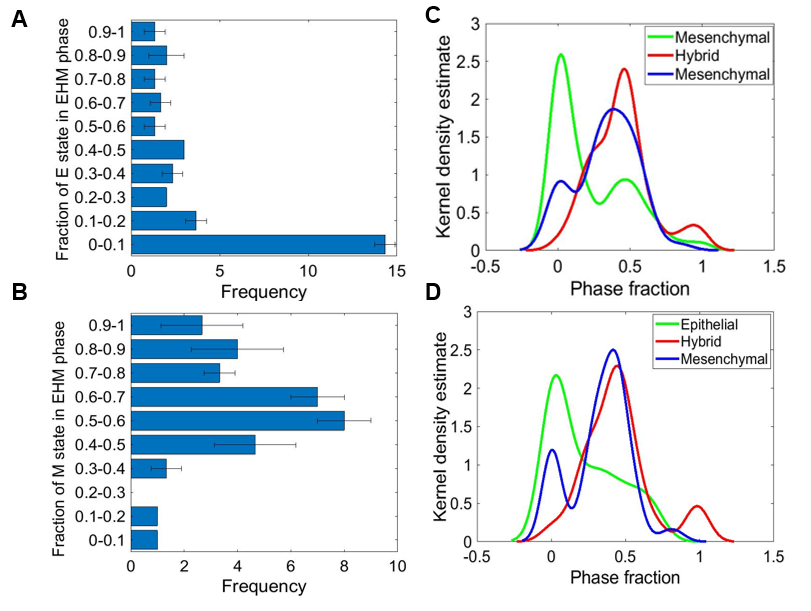


***FigS7: Relative stability analysis****. A) Frequency distribution of E state in the {E,H,M} phase. (B) Frequency distribution of M state in the {E,H,M} phase. C-D) Density plot showing the distribution of E, H and M state in the {E,H,M} phase for two parameter sets obtained from two independent RACIPE replicates/runs.*


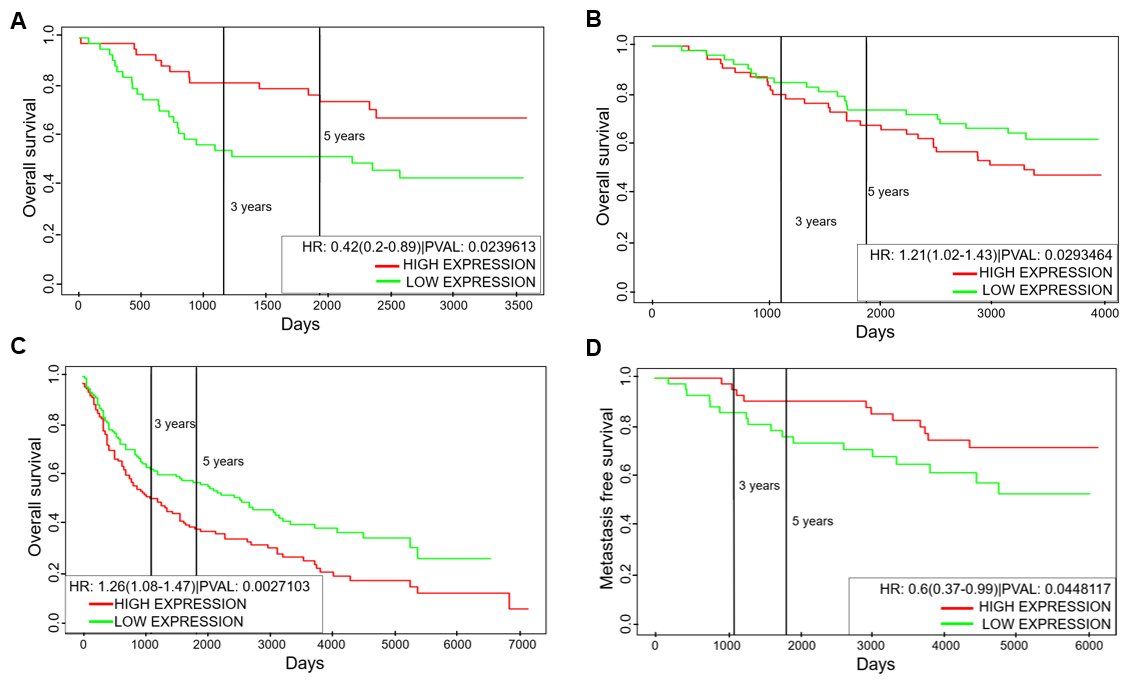


**FigS8: *Clinical outcome of NFATc1 levels is tissue dependent****. (A) Overall survival for GSE14814 (Lung cancer sample). B-C) Overall survival for GSE19536 (Breast cancer samples) and GSE30219 (Lung cancer samples). D) Metastasis free survival for GSE6532 (breast cancer samples). Group of patients with high NFATc1 is denoted by red curve; those with low NFATc1 is depicted by green curve. All cohorts are divided based on median levels.*

**References:**

1. Lu M, Jolly MK, Levine H, Onuchic JN, Ben-Jacob E. MicroRNA-based regulation of epithelial-hybrid-mesenchymal fate determination. *Proc Natl Acad Sci U S A* (2013) **110**:18174–9. doi:10.1073/pnas.1318192110

2. Mooney SM, Jolly MK, Levine H, Kulkarni P. Phenotypic plasticity in prostate cancer: role of intrinsically disordered proteins. *Asian J Androl* (2016) **18**:704–10. doi:10.4103/1008-682X.183570

3. Gould R, Bassen DM, Chakrabarti A, Varner JD, Butcher J. Population Heterogeneity in the Epithelial to Mesenchymal Transition Is Controlled by NFAT and Phosphorylated Sp1. *PLoS Comput Biol* (2016) **12**:e005251. doi:10.1371/journal.pcbi.1005251

4. Oikawa T, Nakamura A, Onishi N, Yamada T, Matsuo K, Saya H. Molecular and Cellular Pathobiology Acquired Expression of NFATc1 Downregulates E-Cadherin and Promotes Cancer Cell Invasion. (2013) doi:10.1158/0008-5472.CAN-13-0274

5. Serfling E, Chuvpilo S, Liu J, Höfer T, Palmetshofer A. NFATc1 autoregulation: a crucial step for cell-fate determination. *Trends Immunol* (2006) **27**:461–469. doi:10.1016/j.it.2006.08.005
